# Physical and Numerical Simulations of Closed Die Hot Forging and Heat Treatment of Forged Parts

**DOI:** 10.3390/ma14010015

**Published:** 2020-12-22

**Authors:** Łukasz Poloczek, Łukasz Rauch, Marek Wilkus, Daniel Bachniak, Władysław Zalecki, Valeriy Pidvysotsk’yy, Roman Kuziak, Maciej Pietrzyk

**Affiliations:** 1 Łukasiewicz Research Network, Institute for Ferrous Metallurgy, ul. K. Miarki 12, 44-100 Gliwice, Poland; wzalecki@imz.pl (W.Z.); vpidvysotskyy@imz.pl (V.P.); rkuziak@imz.pl (R.K.); 2 Department of Applied Computational Science and Modelling, AGH University of Science and Technology, al. Mickiewicza 30, 30-059 Kraków, Poland; lrauch@agh.edu.pl (Ł.R.); mwilkus@agh.edu.pl (M.W.); bachniak@agh.edu.pl (D.B.); mpietrz@agh.edu.pl (M.P.)

**Keywords:** closed die forging, hot forging, heat treatment, physical simulation, numerical simulation

## Abstract

The paper describes physical and numerical simulations of a manufacturing process composed of hot forging and controlled cooling, which replace the conventional heat treatment technology. The objective was to investigate possibilities and limitations of the heat treatment with the use of the heat of forging. Three steels used to manufacture automotive parts were investigated. Experiments were composed of two sets of tests. The first were isothermal (TTT) and constant cooling rate (CCT) dilatometric tests, which supplied data for the identification of the numerical phase transformation model. The second was a physical simulation of the sequence forging-cooling on Gleeble 3800, which supplied data for the validation of the models. In the numerical part, a finite element (FE) thermal-mechanical code was combined with metallurgical models describing recrystallization and grain growth during forging and phase transformations during cooling. The FE model predicted distributions of the temperature and the austenite grain size in the forging, which were input data for further simulations of phase transformations during cooling. A modified JMAK equation was used to calculate the kinetics of transformation and volume fraction of microstructural constituents after cooling. Since the dilatometric tests were performed for various austenitization temperatures before cooling, it was possible to include austenite grain size as a variable in the model. An inverse algorithm developed by the authors was applied in the identification procedure. The model with optimal material parameters was used for simulations of hot forging and controlled cooling in one of the forging shops in Poland. Distributions of microstructural constituents in the forging after cooling were calculated. As a consequence, various cooling sequences during heat treatment could be analyzed and compared.

## 1. Introduction

Problems of the heat treatment of hot forged parts have been intensively studied and numerous papers have been published; see, for example, [1,2,3] or a paper dedicated to automotive parts [4]. On the other hand, due to its constraints, the heat treatment of automotive parts with the use of the heat of forging is still a challenge and published material is less numerous. Advantages of this process are discussed in [5]. They can be summarized as follows: (i) hot forgings with outstanding mechanical properties can be produced, (ii) economic and ecological advantages can be achieved in contrast to conventional hot forging process chains, which consist of various heating and cooling cycles, and (iii) different forging properties and microstructures can be set up by an adjusted temperature control. The majority of publications dealing with this topic are focused on experiments. Some papers consider annealing before forging [6]. The authors of [7] provided general information on direct-forge quenching (DFQ) and direct heat treatment (DHT) processes that are used in automotive and other mechanical industries. Technological advances in these processes and their ability to produce high-quality components at a low production cost were discussed. A spray quenching unit has been designed in [7] for integrated heat treatment in the forging process chain for steel components. Several quenching strategies with varying process conditions have been simulated based on time-temperature-transformation (TTT) diagrams. The design of a quenching unit for the control of the microstructural evolution was based on the simulation results. Few papers have dealt with evaluation of the effects of hot forging and post-forging heat treatment on the mechanical properties of products [8]. Authors of [9] designed a spray quenching unit for integrated heat treatment in the forging process chain for steel components. The simulations helped the design of a quenching unit integrating a sensor device for the control of the microstructural evolution in the workpieces during the quenching. The possibility to perform homogeneous bainite transformation from the forging heat using a stepped quenching strategy (combining gas jets and sprays as well as isothermal heat treatment) was demonstrated for such types of parts as a stepped shaft and a common rail. Authors of [10] used a finite element (FE) model in a macro scale and a material model based on the additive strain decomposition in the micro scale.

In a majority of the discussed papers, constant cooling rate (CCT) and/or isothermal (TTT) diagrams were used to predict phase transformations. Exploring the possibility of more reliable and still fast modelling of phase transformations during complex cooling schedules after hot forging was the motivation of the present work. A review of phase transformation models [11] divides these models into two groups: mean field models and full field models. The latter are based on such methods as phase field, Cellular Automata or Monte Carlo. As long as the latter models account for the geometrical features of the material microstructure, the former describes metallurgical phenomena in a single material point using closed-form equations. Since the hot forging process involves significant heterogeneity of strains, stresses and temperatures, the finite element (FE) method is needed to describe macro scale phenomena. Accounting for the influence of these heterogeneities on gradients of properties in the final product requires a solution of the phase transformation model in each Gauss point of the FE mesh [12], which practically eliminates the full field models. Thus, the emphasis in the present work was put on the upgrade of the mean field models to extend their predictive capabilities while the computing costs remain very low.

The general objective of the present paper was to further investigate possibilities and limitations of heat treatment with the use of the heat of forging. This goal was reached by both numerical and physical simulations of the process. The particular focus was on development of a new, reliable and fast numerical multiscale model of the thermal, mechanical and metallurgical phenomena during hot forging and cooling. This was done by combining basic material tests with physical and numerical simulations of the manufacturing cycle composed of hot forging and controlled cooling.

## 2. Model

### 2.1. FE Model

The finite element (FE) program Forge NxT 3.0 (version number) developed by Transvalor [13] was used in simulations of thermal and mechanical phenomena in the macro scale during forging and cooling. Meshing was calculated using the package’s tool from the curvature of initial mesh. The parts were re-meshed between stages. However, since the geometry changes significantly during forging, periodic remeshing every 20 computational steps was applied to avoid numerical errors. To make computations faster, tool geometries were simulated as contact surfaces.

During forging, the time step was set by built-in routines, which adapt the step according to previous deformation or temperature results. However, since during the cooling simulation the recalescence heat is returned to the solver, a different time step adaptation scheme was used in the thermal simulation. A standard adaptation based on previous values was used until the recalescence heat became non-zero. Then, a reduced time step was introduced as a constant value set, used for the entire length of transformations.

The heat transfer coefficient for the air cooling was taken from program’s database. For accelerated cooling on the cooling line, the coefficient was identified by comparing the simulated temperature profile with measurements. The material models supplied in the Forge^®^ package were used in mechanical (flow stress) and thermal (conductivity, specific heat, density, expansion coefficient) parts. The flow stress, which is the material parameter in the mechanical part, was calculated based on the Hansel-Spittel equation with coefficients, which were identified using inverse analysis for the hot compression tests results. The inverse algorithm described in [11] was used. Equations describing phase transformations during cooling were implemented in the user’s procedures in the FE program.

The objectives of the FE simulations were twofold. The first was calculation of the strain, strain rate and temperature history in the selected locations in the forging. This history was reproduced in the physical simulation on the Gleeble 3800. Simulations of the distribution of the microstructural parameters in the whole volume of the forgings was the second objective.

### 2.2. Microstructure Evolution Model

Equations describing processes of recrystallization and grain growth during hot forming were based on the fundamental works of Sellars at the University of Sheffield [14], and these equations are in the Forge database. Coefficients in the equations for the investigated steels were determined in the present project on the basis of compression tests, stress relaxation tests and grain growth simulations (see chapter 4). All equations are given in Table 1, where: SRX—static recrystallization, DRX—dynamic recrystallization, *t*—time, *ε*—strain, $\dot{\varepsilon }$—strain rate, *T*—temperature in K, *R—*gas constant, *D*_0_—grain size prior to deformation, Z—Zener-Holomon parameter, *Q_SRX_*, *Q_DRX_*, *Q_DSRX_*, *Q_GROWTH_*—activation energy for the relevant process, *n*, *a*_0_−*a*_3_, *b*_0_−*b*_3_, *p*_1_−*p*_10_, *q*, *K*—coefficients determined by the inverse analysis of the experimental tests.

The FE model with microstructure evolution equations implemented into the FE Forge code predicted distributions of the temperature and the grain size in the part after hot forging. These data were a starting point for further simulations of phase transformations during cooling. 

### 2.3. Phase Transformation Model

The modified JMAK (Johnson-Mehl-Avrami-Kolmogorov) equation was used to calculate the kinetics of transformation and volume fraction of microstructural constituents after cooling. The main equation of this model is

(8) $$X=1-\exp\left(-kt^{n}\right)$$

where: *X—*volume fraction of a new phase, *t*—time, *k*, *n*—coefficients.

The numerical implementation of this model is described in [15]. Since the dilatometric tests were performed for various austenitization temperatures before cooling, it was possible to account for the effect of the austenite grain size on the kinetics of transformations. The austenite grain size at the beginning of transformations (*D_γ_*) was included as a variable in the model. The main equations in the model are given in Table 2. The following upgrades were introduced in the JMAK equation [16]:

(1) Coefficient *n* is constant for each transformation and in the identification it is referred to as *a*_4_, *a*_16_ and *a*_24_ for ferrite, pearlite and bainite transformations, respectively.
(2) According to [15], coefficient *k* is temperature dependent. The modified Gauss function proposed in [17] was used.
(3) Calculations of carbon concentration in the austenite during both ferrite and bainite transformations were added.
(4) The *T*_0_ temperature concept proposed in [18] was added. It allowed prediction of the return of the pearlitic transformation after bainitic has started during holding at the constant temperature. Beyond this, prediction of the occurrence of the retained austenite became possible.

Equations used for the calculation of the current carbon content in the austenite (*c_γ_*), temperatures of bainite start (*B_s_*) and martensite start (*M_s_*), as well as martensite volume fraction (*F_m_*) are shown in Table 2, where *F_f_*, *F_p_* and *F_b_* represent volume fractions of ferrite, pearlite and bainite, respectively. Parameter *p* in this table is explained in [19] and the numerical solution of the present model is described in [20].

The phase transformation model allows the effect of the recalescence to be taken into account. The heat generated during transformation per unit volume is calculated as

(18) $$Q=\rho H\frac{dX}{dt}$$

where: *ρ—*density, *H—*enthalpy of the phase transformation. 

The phase transformation model was implemented into the FE Forge software using a user subroutine. The model was compiled to library form and connected with the FE package using its user routines. This technique allows the single implementation to be used in various FE programs only by adapting interface characteristics to the specific application. Source data is obtained from computation, intermediate variables are stored in mesh and results are passed as typical software-specific fields to be post-processed or used in other routines. In Forge software, two additional routines were added to obtain model coupling:

(1) A routine which prepares storage for the model’s intermediate variables, initializes or obtains simulation parameters passed to the model and calls the model main routine from a library file. The results are similarly stored in mesh fields and can be used in further simulation steps.
(2) A routine which converts model results, especially transformation energy, to values accepted by the solver’s FE thermal coupling (in the analysed case, thermal power). This routine allows a full thermal coupling of transformation on an energy level to be obtained without using additional models to compensate the transformation with modifying material parameters and can be turned off to make computations faster, but ignores thermal coupling.

Coupling of the FE code with the phase transformation model allowed for feedback from the micro scale to macro scale. As a consequence, the kinetics of transformations was calculated for the current, local temperatures, and the heat generated in the microstructure due to recalescence, calculated from Equation (18), was used in the macro FE calculations of the temperature.

## 3. Experiments

### 3.1. Methodology

Uniaxial compression tests supplied data for identification of the flow stress model, and stress relaxation tests supplied data for identification of the microstructure evolution model. All the tests were carried on Gleeble 3800. The cylindrical samples having dimensions Ø10 mm × 12 mm were compressed at different temperatures, strain and strain rates. The methodology of these tests is well known and is not described here. 

Dilatometric experiments were performed with a DIL 805 A/D/T dilatometer (New Castle, DE, USA). The experiments were intended to supply isothermal (TTT) and constant cooling rate (CCT) phase transformations diagrams, which were the input data for identification of phase transformation models. Prior to the cooling stage of the experiment, the samples (Ø5 mm × 10 mm) were subject to the deformations reproducing the forging process. 

The physical simulation of the forging-cooling sequence was carried out on the Gleeble 3800 (Poestenkill, NY, USA) using blocky samples having dimensions 15 mm × 20 mm x 35 mm, which were subject to three-stage compression. Strain, strain rate and temperature variations calculated using finite element (FE) code Forge were reproduced in the experiments. This history for the two representative points in each forging (one located in a massive part and the second in the thin part of the forging) was reproduced on the Gleeble 3800. The physical simulations supplied data for the validation of the models and were used to design the industrial cooling sequence. 

The microstructural observations were performed with light optical-digital microscopy (LOM) using an Olympus DSX500i (Tokyo, Japan) and with scanning electron microscopy (SEM) using INSPECT F (Tokyo, Japan). Quantitative analysis of grain size and volume fractions was carried out using MetiIo software. Quantitative evaluation of grain size was done on 10 micrographs recorded with 1000 times magnification, using an LOM microscope. In turn, the quantitative analysis of volume fractions of structural components was carried out on 20 micrographs recorded with 2000 times magnification, using the SEM microscope.

### 3.2. Materials

Manufacturing of three products, subject to different heat treatment involving controlled cooling after hot forging, was considered:

(1) adapter made of 42CrMo4 steel (steel A in Table 3) subject to isothermal annealing,
(2) flange made of the C22.8 steel (steel B in Table 3) subject to normalization,
(3) fork made of the C45 steel (steel C in Table 3) subject to quenching and tempering.

These steel grades were chosen in the present investigation due to their diversified behaviour during forging and subsequent cooling.

### 3.3. Deformation and Thermal Cycles

Dilatometric tests were performed for various austenitization parameters; as a consequence, the effect of the austenite grain size after forging on the kinetics of transformation could be included in the model. The cooling rates varied between 0.1 °C/s and 100 °C/s, which covered different investigated controlled cooling processes. 

### 3.4. Physical Simulations

**Figure 1:**
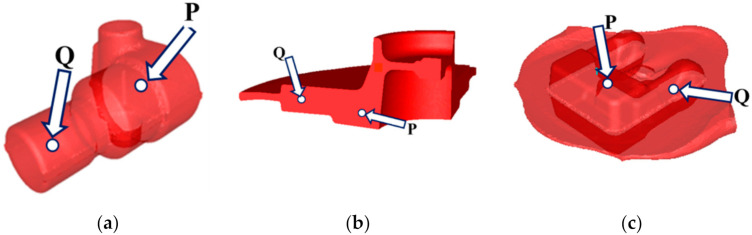
Schematic illustration of the investigated forgings with the locations of sensors: (**a**) adapter, (**b**) flange, (**c**) fork.

Physical simulations covered the whole process including three step forging and controlled cooling. As has been mentioned, two characteristic points in each forging were selected. One point (P) was located in the massive part of the forging and the second (Q) in the thinnest part. Schematic illustration of the investigated forgings with the locations of sensors is shown in Figure 1. Strain and temperature history, calculated with the Forge program, in these points during forging and cooling were reproduced on the Gleeble 3800 thermomechanical simulator. The samples were water quenched at various stages of the cooling process. The microstructure of the samples was analysed after each test and volume fractions of phases were estimated.

Time-deformation-temperature data for the physical simulation are given in Table 4. The temperatures in this table refer to the beginning of the deformation. The strain rate varied during deformations and the maximum value was about 25 s^−1^.

**Figure 2:**
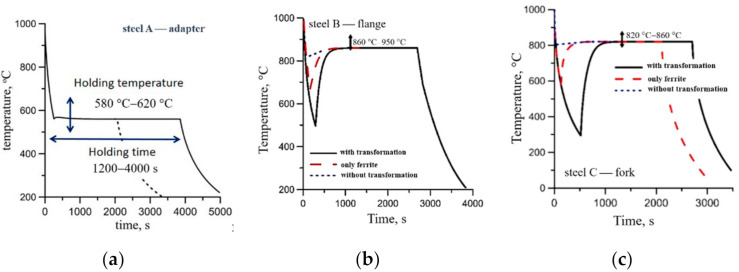
Thermal cycles in points P during cooling of the investigated forgings: (**a**) annealing of the adapter, (**b**) normalization of the flange, (**c**) quenching and tempering of the fork.

Variations of the temperature versus time during cooling of the samples subject to the three-stage deformation on Gleeble 3800 are shown schematically in Figure 2. The following three variants of cooling before holding were considered:

(1) Variant 0—cooling to the temperature above *A_c_*_3_, heating to the holding temperature (all steels).
(2) Variant 1—cooling until the austenite decomposition is completed, heating to the holding temperature (steels B and C).
(3) Variant 2—cooling until the ferritic transformation is completed and pearlitic transformation did not begin, heating to the holding temperature (steels B and C).

The objective was to investigate the effect of the microstructure at the beginning of holding on the phase transformations.

Holding time for steel A varied between 1800 s and 3600 s. The objective was to investigate progress of pearlitic and bainitic transformations during cooling. The holding time for steels B and C was 1 h.

### 3.5. Results for Steel A

**Figure 3:**
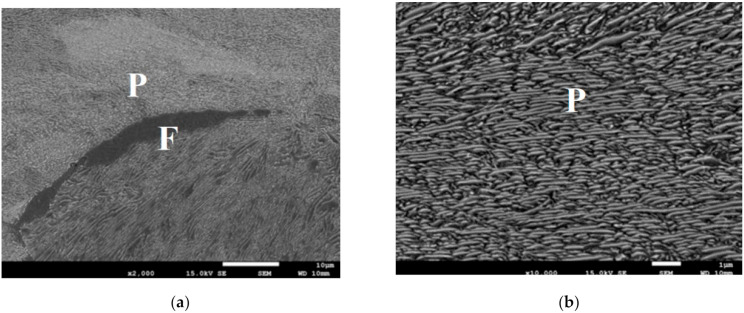
(**a**,**b**) Selected microstructures of the samples after holding for 1 h at the temperature of 620 °C, various magnifications. P—pearlite; F—ferrite.

Grain size at the beginning of transformations was at the level of 50 ± 4 μm for the massive part of the forging and 40 ± 3 μm for the thinner part. Due to the small difference, all the results presented below are for the massive part only. In the tests with the holding temperature above 600 °C, pearlitic microstructures, with some ferrite at the level of around 5%, were obtained. Only one example of the microstructure of the pearlite for the holding temperature 620 °C is presented in Figure 3, and these results are not reproduced further in the paper. A more complex situation was observed at lower temperatures, in which bainitic transformation was dominating but it was controlled by the *T*_0_ line. 

**Figure 4:**
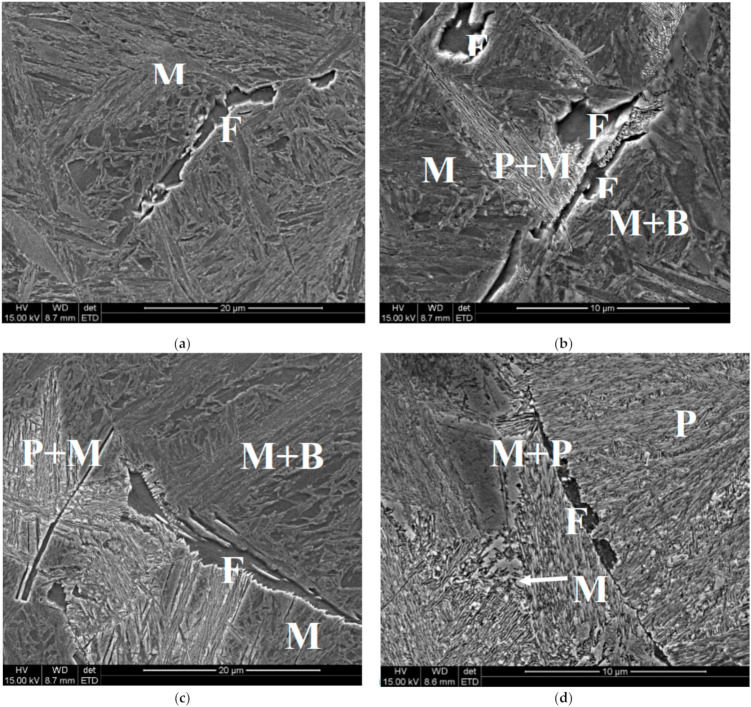
Microstructures of the samples after holding at the temperature of 560 °C for 600 s (**a**), 1200 s (**b**), 2400 s (**c**) and 4000 s (**d**) F—ferrite, P—pearlite, B—bainite, M—martensite.

**Figure 5:**
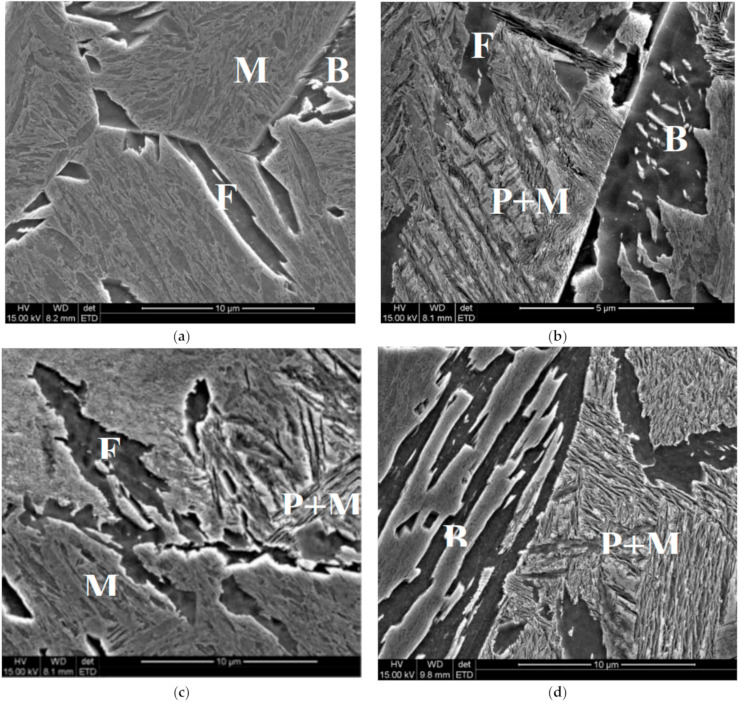
Microstructures of the samples after holding at the temperature of 520 °C for 600 s (**a**), 1000 s (**b**), 2000 s (**c**) and 4000 s (**d**). F—ferrite, P—pearlite, B—bainite, M—martensite.

Microstructures of the samples after holding at temperatures of 560 °C and 520 °C are shown in Figure 4 and Figure 5, respectively. Analysis of the pictures for 520 °C allows suggestion that pearlitic and bainitic transformations could occur alternately, however, the bainitic transformation occurs first and proceeds the pearlitic one until the moment when carbide content in untransformed austenite reaches the *T*_0_ line. The possibility of modelling of this phenomenon is described in [19], and this approach was applied in the present work. Microstructures show also that even for very long holding times the austenite decomposition is not completed and the remaining austenite is transferred into bainite or martensite during subsequent cooling. Volume fractions of phases were determined for each sample and these results are presented in Section 5.1, where they are compared with the model predictions.

### 3.6. Results for Steel B

**Figure 6:**
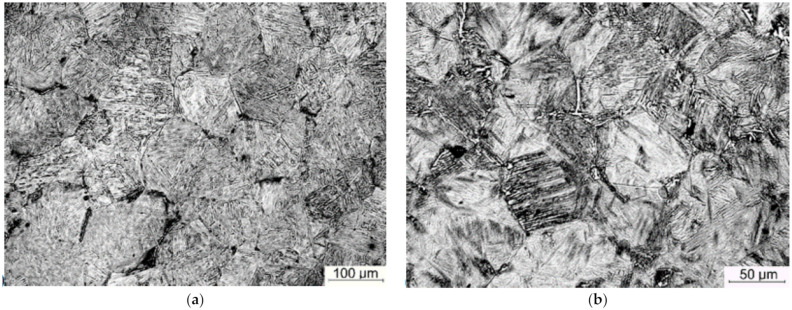
Microstructures of steel B before the first deformation (**a**) and after the last deformation (**b**).

Physical simulations involved three step deformation followed by cooling according to schedules shown in Figure 2b. The microstructures in point P before the first deformation and after the last deformation are shown in Figure 6. In this case, the samples were cooled with water prior to and after deformations. The microstructure in that case contains thin ferrite layers decorating austenite grain boundaries and martensite + bainite mixture inside grains. The measured austenite grain size was 90 ± 7μm and 50 ± 4 μm, respectively. The grain size was similar in both points P and Q.

**Figure 7:**
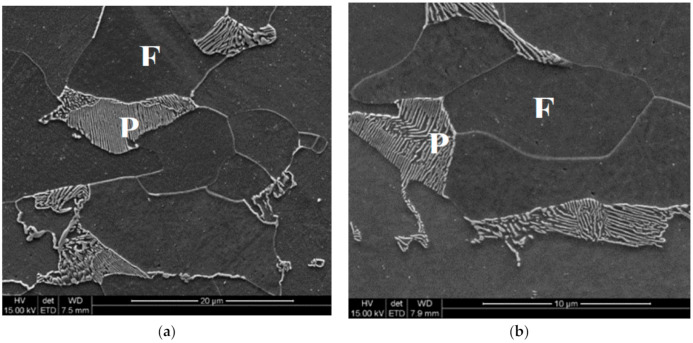
Microstructures of steel B in point P after cooling variant 1 (**a**) and variant 0 (**b**). F—ferrite, P—pearlite.

Three cooling sequences were simulated. The difference was in the lowest temperature after the first cooling following forging. This temperature was 344 °C, 697 °C and 726 °C for point P and 344 °C, 662 °C and 697 °C for point Q, for variants 1, 2 and 0, respectively. The temperatures were selected to obtain complete austenite decomposition (variant 1), only ferritic transformation without pearlite (variant 2) and less than 20% of the ferritic transformation (variant 0) in point P. The temperatures in point Q were slightly lower. The holding temperature was 920 °C for all variants. Cooling in air was applied after the holding, and the cooling rate varied between 3 °C/s at the beginning of cooling and below 1 °C/s at the end of cooling. Selected microstructures after cooling are shown in Figure 7. Quantitative analysis of the microstructural constituents is presented in Section 4.

### 3.7. Results for Steel C

**Figure 8:**
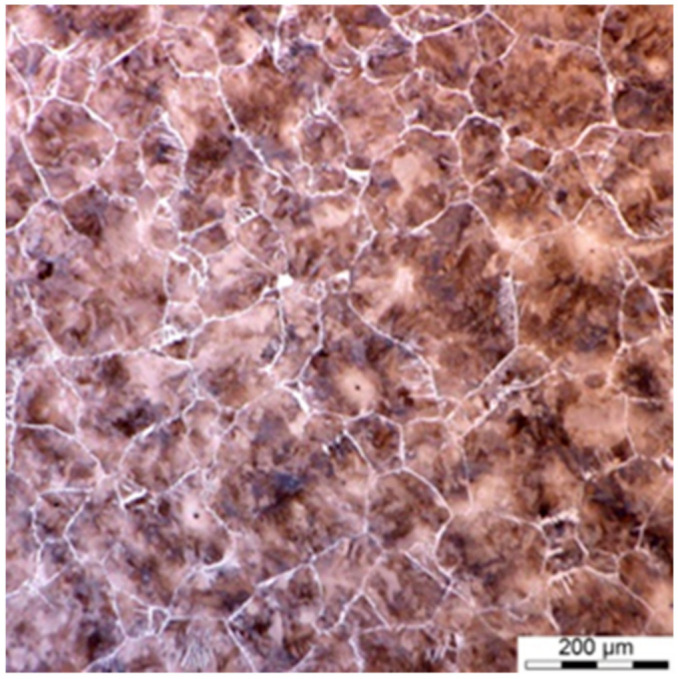
Microstructures of steel C before the first deformation.

Physical simulations involved four step deformation followed by cooling according to the schedules shown in Figure 2c. The microstructure in point P before the first deformation is shown in Figure 8. As in the case of A steel, fast cooling was applied to cause the precipitation of ferrite at the austenite grain boundaries. The grain size was similar in both points. It was non-uniform, with an average value of 100 ± 12 μm.

**Figure 9:**
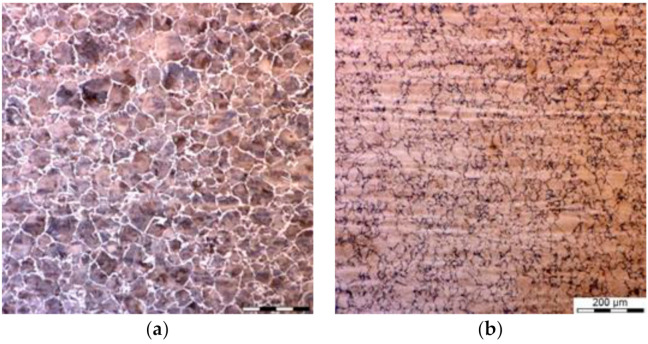

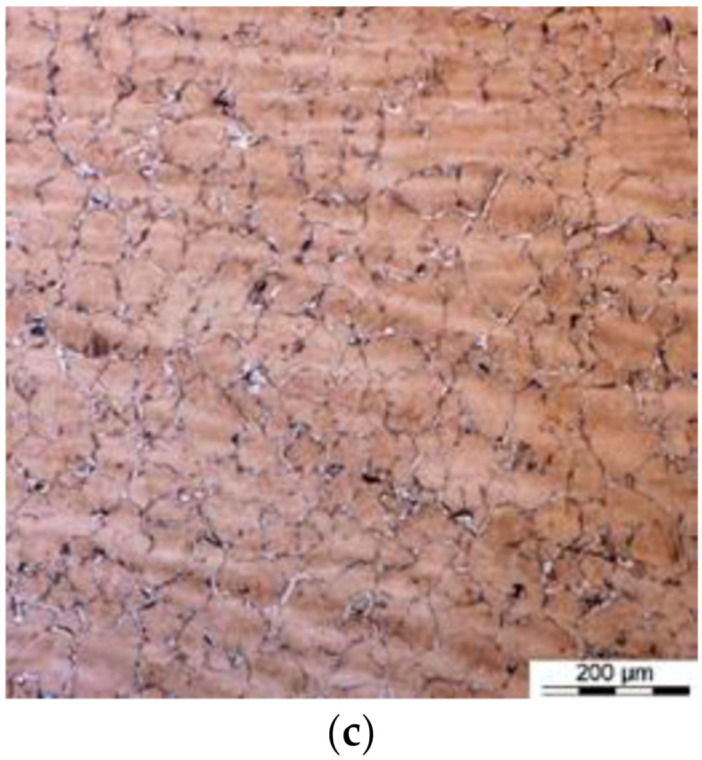
Point P variant 1—microstructures of the samples after first cooling to 540 °C (**a**), after heating to the holding temperature (**b**) and at the end of holding (**c**).

**Figure 10:**
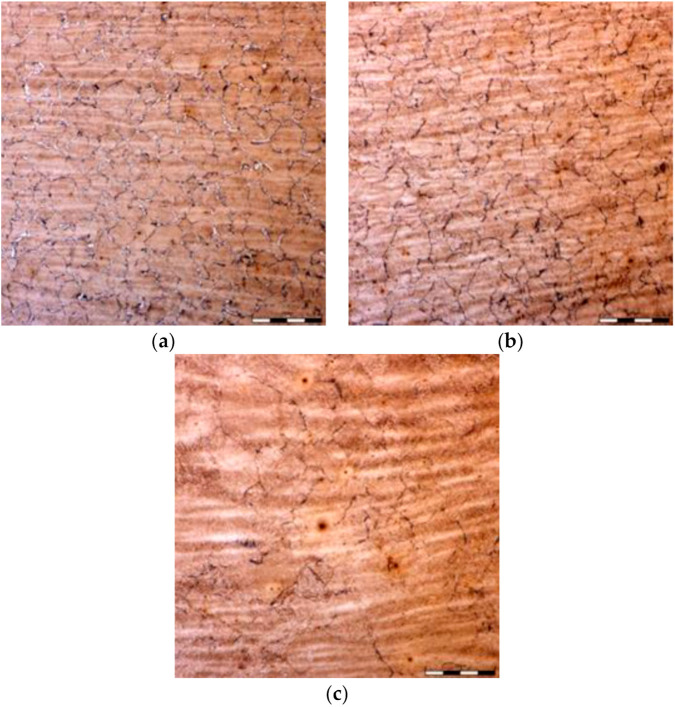
Point P variant 2—microstructures of the samples after first cooling to 670 °C (**a**), after heating to the holding temperature (**b**) and at the end of holding (**c**).

The results for cooling are presented for the holding temperature of 860 °C. The first set of results concerns point P in Figure 1c. Grain size during cooling according to the variant 0 was 70 ± 3 μm at the beginning of holding and 74 ± 4 μm at the end of holding. Microstructures during cooling according to variants 1 and 2 are shown in Figure 9 and Figure 10. Microstructure after the first cooling to 540 °C (variant 1) is shown in Figure 9a. It is composed of about 12% of ferrite and pearlite. Ferrite and pearlite transferred into austenite during heating and fully austenitic microstructures at the beginning and end of holding were obtained. During further cooling the austenite grain boundaries were decorated by ferrite grains, which is seen in Figure 9b,c for the beginning and end of holding, respectively. The measured austenite grain size was 39 ± 2 μm in Figure 9b and 45 ± 2 μm in Figure 9c.

The microstructure in point P after the fast cooling to 670 °C is shown in Figure 10a. It is composed of about 5% of pearlite and ferrite. Fully austenitic microstructures at the beginning and end of holding are shown in Figure 10b,c, respectively. The measured austenite grain size was 63 ± 3 μm and 85 ± 5 μm.

**Figure 11:**
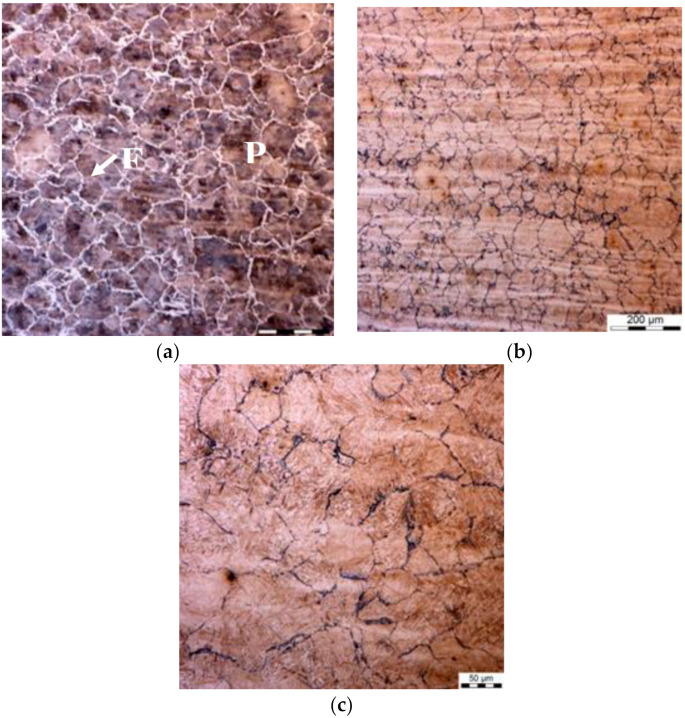
Point Q variant 1—microstructures of the samples after first cooling to 500 °C (**a**), after heating to the holding temperature (**b**) and at the end of holding (**c**).

**Figure 12:**
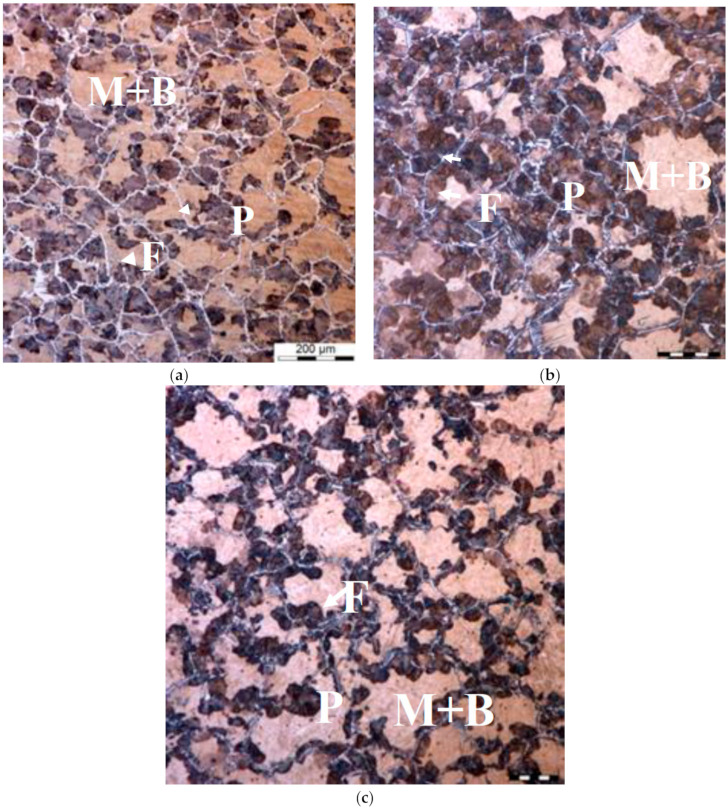
Point Q variant 2—microstructures of the samples after first cooling to 609 °C (**a**), after heating to the holding temperature (**b**) and at the end of holding (**c**).

Similar results were obtained for point Q in Figure 1c. Grain size during cooling according to the variant 0 was 83 ± 5 μm at the beginning of holding and 87 ± 4 μm at the end of holding. The differences between austenite grain size after reheating and holding are not significant, which means that the grain growth slowed down after reaching the holding temperature. Microstructures during cooling according to variants 1 and 2 are shown in Figure 11 and Figure 12. The microstructure after the first cooling to 500 °C (variant 1) is shown in Figure 11a. It is composed of ferrite (about 14%). Fully austenitic microstructures at the beginning and end of holding are shown in Figure 11b,c, respectively. The measured austenite grain size was 54 ± 3 μm and 61 ± 5 μm.

The microstructure in point Q after the first cooling to 609 °C followed by fast cooling in water is shown in Figure 12a. It is composed of about 40% of pearlite, 10% of ferrite and martensite/bainite, which was formed during water cooling. Microstructures at the beginning and end of holding are shown in Figure 12b,c, respectively. It is seen that pearlite transformation continued during heating prior to holding. On the contrary, the rate of cooling after holding was too small to prevent against the pearlite formation. Despite this, austenite grain size could be estimated by the measurement conducted on that sample. The measured austenite grain size was, respectively, 55 ± 3 μm and 71 ± 2 μm.

**Figure 13:**
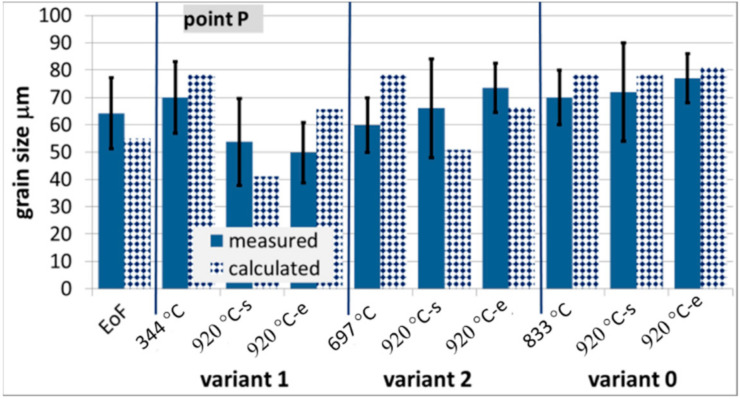
Measured and calculated austenite grain size at various stages of the process for steel B, EoF—end of forging.

It is noteworthy that the fraction of pearlite in the sample reheated to the holding temperature contains more pearlite, which means that the austenite kept decomposing to this phase during heating of the sample (Figure 13).

## 4. Identification and Verification of the Austenite Microstructure Evolution Model

An inverse algorithm developed by the authors [11] was applied in the identification procedure. Identification of the microstructure evolutions model in Table 1 was divided into two parts. A static recrystallization model was identified on the basis of stress relaxation tests [21]. A dynamic recrystallization model was identified on the basis of compression tests at various temperatures and various strain rates. The coefficients in the microstructure evolution model for the three steels are given in Table 5.

Identification of the phase transformation model was performed using inverse analysis for the dilatometric tests. In the analysis, the phase transformations start and finish temperatures, as well as volume fractions of structural constituents, determined in the dilatometric experiments, were used. Basic principles of application of this method to phase transformation models are described in [22]. In a majority of earlier applications of this method, results of the constant cooling rate (CCT) tests only were satisfactory to obtain reliable results. In the present project, however, heat treatment processes involve holding at the constant temperature in the range of pearlitic or bainitic transformation. Therefore, to improve the identification, the results of both CCT and TTT tests were combined. The following objective function was used:

(19) $$\Phi =\underset{a}{\underbrace{\min}}\left[\sqrt{w_{T}\frac{1}{k}\overset{k}{\underset{i=1}{\sum }}{\left(\frac{T_{m,i}-T_{c,i}}{T_{m,i}}\right)}^{2}+w_{F}\frac{1}{l}\overset{l}{\underset{i=1}{\sum }}{\left(\frac{F_{m,i}-F_{c,i}}{F_{m,i}}\right)}^{2}+w_{t}\frac{1}{m}\overset{m}{\underset{i}{\sum }}{\left(\frac{t_{m,i}-t_{c,i}}{t_{m,i}}\right)}^{2}}\right]$$

where: *w_T_*, *w_F_*, *w_t_—*weights, *T_m_*, *T_c_—*measured and calculated start and end temperatures of phase transformations in the CCT tests, *F_m_*, *F_c_—*measured and calculated volume fractions of phases, *t_m_*, *t_c_*—measured and calculated times to the start and end of phase transformations in the TTT tests, *k*, *l*, *m*—number of temperatures, volume fractions and times, respectively.

The identification of the phase transformation model was performed using high performance computing (HPC) infrastructure. It was dictated by a large number of model coefficients and, as a consequence, requires huge computational costs to obtain reliable model. Over one hundred optimizations were performed using 50 nodes, each particular node had 24 computational cores. In this particular problem, global optimization algorithms were applied (PSO and Genetic), using from 24 to 96 specimens. Finally, the best model was selected. This approach allowed problems with local minima to be resolved and finally a reliable model to be obtained.

Coefficients in all models were determined by searching for the minimum of the objective Equation (19) with respect to these coefficients. The results are given in Table 6 for steel A, in Table 7 for steel B and in Table 8 for steel C.

**Figure 14:**
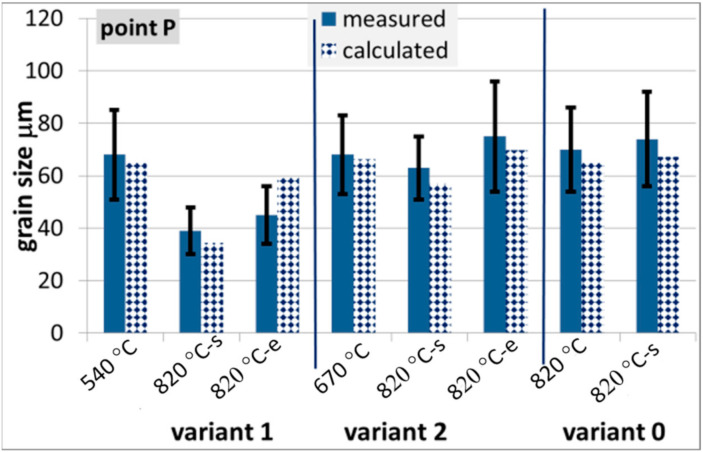
Measured and calculated austenite grain size at various stages of the process for steel C.

The microstructure evolution model was verified via comparison of the measured and calculated austenite grain size at various stages of the process during physical simulations. Selected examples of the results for steels B and C only are presented in Figure 13 and Figure 14.

**Figure 15:**
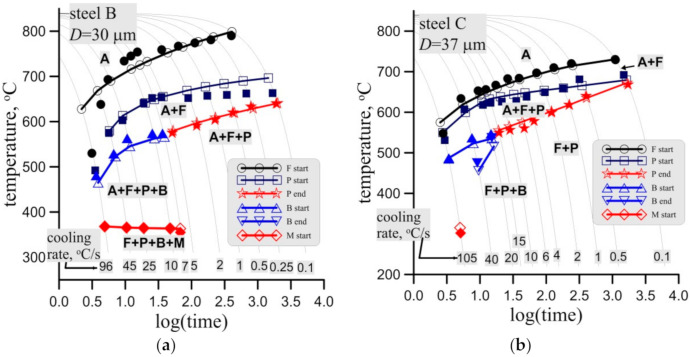

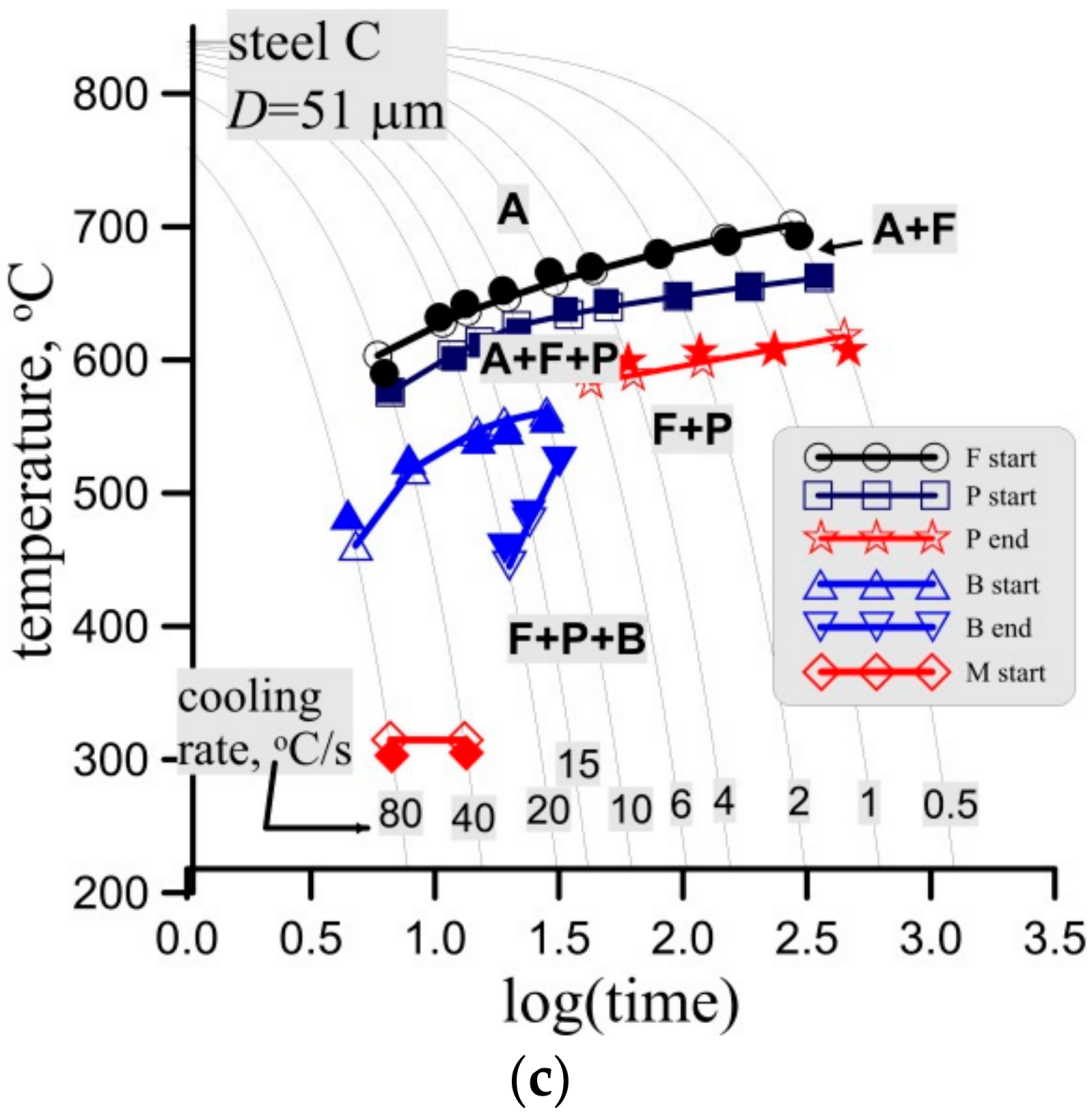
Measured (full symbols) and calculated (open symbols with lines) start and end temperatures of phase transformations in the CCT tests for steel B, grain size 30 ± 3 μm, (**a**) and steel C, grain size 37 ± 4 μm (**a**) and 51 ± 3 μm (**c**).

The quality of the identification of the phase transformation model was evaluated via comparison of measured and calculated start and end temperatures for the phase transformations in the CCT tests and times to the start and end of transformations in the TTT tests. Selected results for steel B and steel C are shown in Figure 15 here. Reasonably good agreement was obtained. It should be noted that one model is used for different austenite grain size prior to transformations.

**Figure 16:**
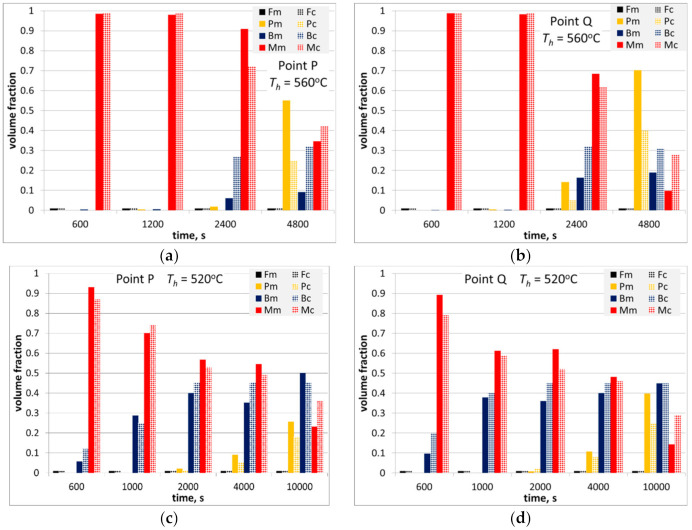
Measured and calculated volume fraction of phases in steel A after physical simulations described in Section 3.3, holding temperature 560 °C (**a**,**b**) and 520 °C (**c**,**d**) in the massive part of the forging (**a**,**c**) and in the thinner part (**b**,**d**).

**Figure 17:**
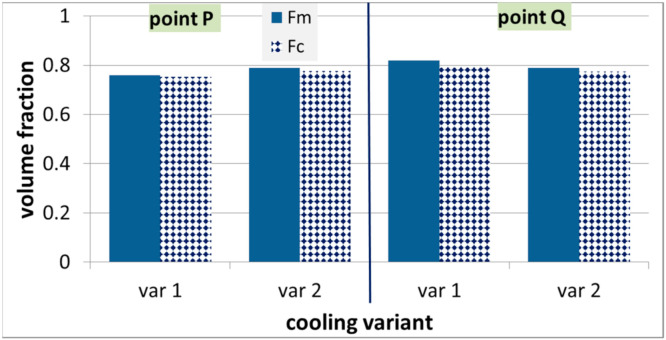
Measured and calculated volume fraction of ferrite in steel B after physical simulations described in Section 3.3.

The model was further verified via comparison of the measured and calculated volume fractions of microstructural constituents after thermal cycles shown in Figure 2. Results of this comparison are shown in Figure 16 and Figure 17 for steels A and B, respectively.

The model predicts very well volume fractions of structural components for ferritic/pearlitic microstructures. Discrepancies are larger when bainite and martensite are dominating. It is caused by much larger magnifications of the microstructure, which were necessary to distinguish these components. As a consequence, the area of analysis was constrained and was less representative for the whole material.

## 5. Numerical Simulation of Forging-Cooling Sequences

The validated model with optimal material parameters was used for simulations of hot forging and controlled cooling in one of the forges in Poland. Distributions of microstructural constituents in the forging after cooling were calculated. Selected results are described in the following sections.

### 5.1. Steel A

**Figure 18:**
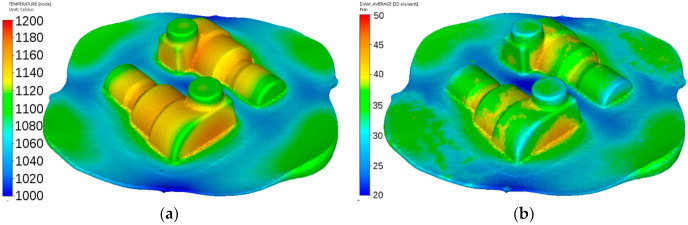
Calculated distributions of the temperature (°C) (**a**) and austenite grain size (μm) (**b**) after forging of the adapter from steel A.

Calculated distributions of the temperature and austenite grain size after forging of the adapter are shown in Figure 18. Temperatures are at the level of 1100–1230 °C, and the austenite grain size varies in the range 30 ± 5 μm in the flash and 70 ± 4 μm in the massive part of the forging. These data were used as a starting point for simulation cooling according to the scheme shown in Figure 2a.

**Figure 19:**
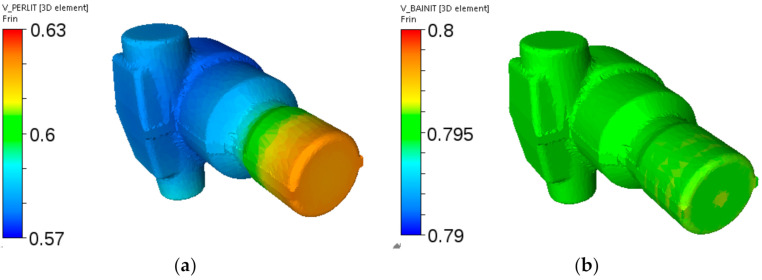
Cooling of the adapter from steel A—calculated distributions of the pearlite volume fraction after holding at 560 °C for 1 h (**a**) and of the bainite volume fraction after holding at 520 °C for 1 h (**b**).

Further simulations were performed for various holding temperatures and holding times, and distributions of phase volume fractions were calculated. Selected results for holding at the temperatures 560 °C and 520 °C are shown in Figure 19. It is seen that pearlite dominates for holding temperature 560 °C and bainite dominates for holding temperature 520 °C. At 560 °C, more pearlite was predicted for the thick end of the forging, which was due to larger austenite grains in that area. At 520 °C, distribution of the bainite volume fraction was more uniform. The martensite was a remaining phase in both cases.

### 5.2. Steel B

**Figure 20:**
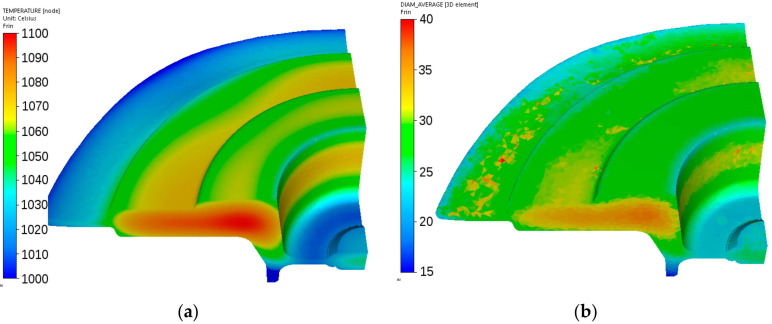
Calculated distributions of the temperature (**a**) and austenite grain size (**b**) after forging of the flange from steel B.

Calculated distributions of the temperature and austenite grain size after forging of the flange are shown in Figure 20. Temperatures are at the level of 950–1110 °C, and the austenite grain size varies in the range of 15 ± 2 μm in the thin part and 45 ± 3 μm in the massive part of the forging. These data were used as a starting point for simulation cooling according to the scheme shown in Figure 2b.

**Figure 21:**
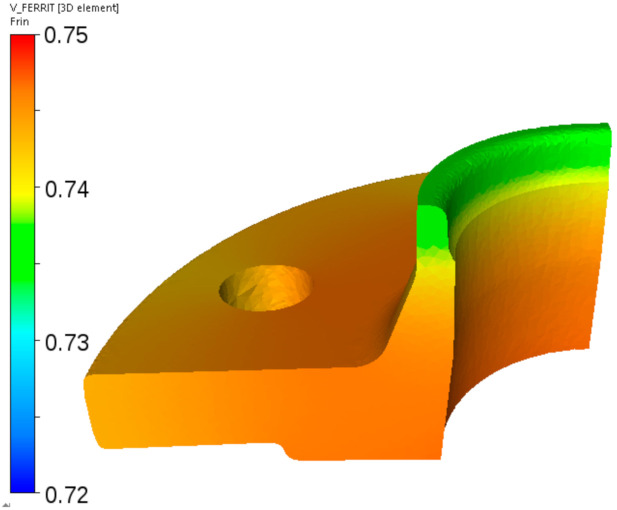
Calculated distributions of the ferrite volume fraction after cooling of the flange from steel B according to the schedule in Figure 2b.

Further simulations were performed for the cooling sequence shown in Figure 2b. Calculated distribution of the ferrite volume fraction after cooling to the room temperature is shown in Figure 21. The level of the ferrite fraction is in the range 72–75%. Pearlite is the remaining phase.

### 5.3. Steel C

#### Calculated Distribution of the Temperature

**Figure 22:**
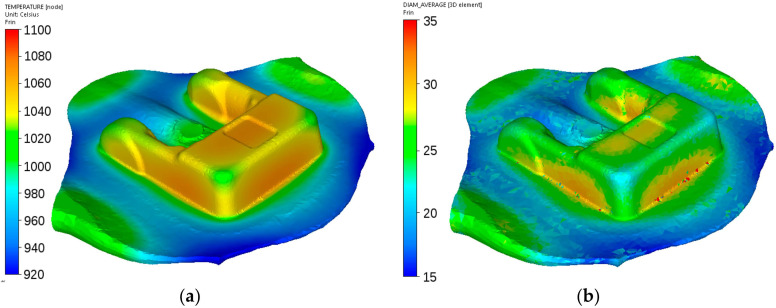
Calculated distributions of the temperature (**a**) and austenite grain size (**b**) after forging of the fork from steel C.

Calculated distributions of the temperature and austenite grain size after forging of the fork are shown in Figure 22. Temperatures are at the level of 940–1130 °C, and the austenite grain size varies in the range 28 ± 5 μm in the arm and 35 ± 4 μm in the massive part of the forging. These data were used as a starting point for simulation of cooling according to the scheme shown in Figure 2c.

**Figure 23:**
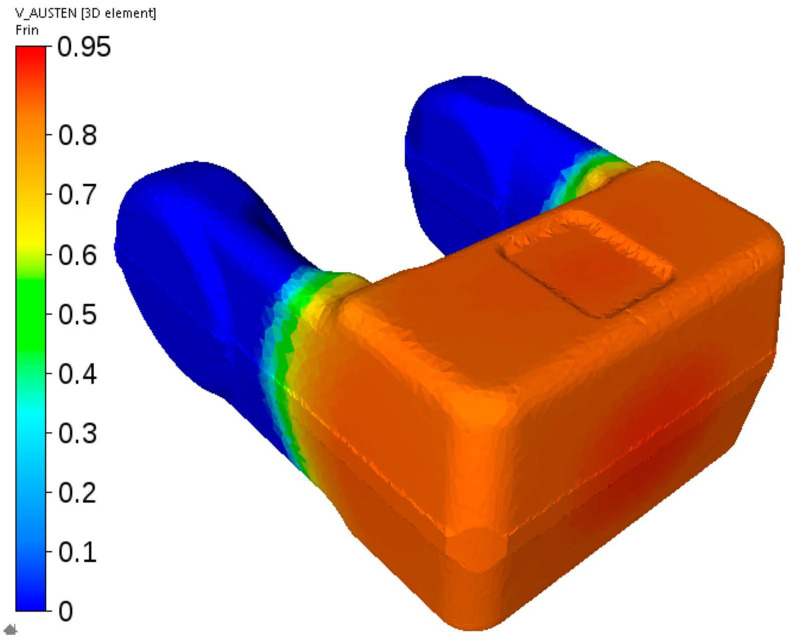
First stage of the cooling of the fork from steel C—calculated distribution of the austenite volume fraction after cooling to the temperature at which ferritic transformation is completed and pearlitic transformation has not begun yet.

The first stage of cooling according to the schedule in Figure 2c was simulated next. Figure 23 shows the calculated distribution of the austenite volume fraction after cooling to the temperature at which ferritic transformation in the arms is completed and pearlitic transformation has not begun yet. It is seen that at this stage in the massive part of the forging the transformation is at the beginning stage and the volume fraction of the remaining austenite is in the range 80–95%.

## 6. Conclusions

The aim of the project was to develop the numerical tools capable of generating alternative cooling routes after forging as compared to conventional heat treatment methods. For this, an upgrade of the JMAK model was developed and applied to simulate various cooling strategies for hot forged parts. The upgrade of the model was composed of:

(1) Introduction of the modified Gauss function to describe the relation of the Avrami coefficient *k* to the temperature.
(2) Introduction of the austenite grain size prior to transformation as an independent variable in the model.
(3) Prediction of the carbon concentration in the austenite during bainitic transformation and using the *T*_0_ line concept to control the progress of the isothermal bainitic transformation.

Dilatometric tests were performed and supplied data for the identification of the phase transformation model for the three steels. Following this, the physical simulations of the selected forging processes were performed and the results were compared with the numerical simulations. The following conclusions were drawn:

(1) The deformation process affects substantially the developed austenite microstructure and affects the kinetics of the decomposition of this phase to ferrite, pearlite and to a lesser extent to bainite and martensite. Therefore, it is important that this effect should be accounted for in the phase transformation model.
(2) The model predicts the start and end temperatures of transformations with good accuracy.
(3) The model predicts very well volume fractions of structural components for ferritic/pearlitic microstructures.
(4) Despite the limitations, the methodology of the model validation and tuning adapted in this investigation using the results of the physical simulation of the forging process with Gleeble 3800 proved to be a very effective tool for improving the predictive capabilities of the mathematical models.

Larger discrepancies between measured and calculated volume fractions occurred when bainite and martensite were dominating components. This can be partly assigned to difficulties with reliable measurements of the bainite and martensite volume fraction. The other phenomenon not accounted for in the model relates to the chemical composition inhomogeneities related to the steel casting process.

Moreover, the global approach adapted in this investigation does not account for local phenomena, specifically those relating to non-equilibrium conditions prevailing at the physical interfaces created during phase transformations. However, this drawback of the developed methodology is counterbalanced by its simplicity and short time of the numerical calculations.

## Figures and Tables

**Table 1:** Equations describing microstructure evolution during hot forming.

Parameter	Equation	
Kinetics of SRX	$X_{SRX}=1-\exp\left[-0.693{\left(\frac{t}{t_{0.5}}\right)}^{n}\right]$ $t_{0.5}=a_{0}\varepsilon^{-a_{1}}{\dot{\varepsilon }}^{a_{2}}D_{0}^{a_{3}}\exp\left(\frac{Q_{SRX}}{RT}\right)$	(1)
Grain size after SRX	$D_{SRX}=b_{0}\varepsilon^{-b_{1}}{\dot{\varepsilon }}^{-b_{2}}D_{0}^{b_{3}}\exp\left(\frac{-Q_{DSRX}}{RT}\right)$	(2)
Critical strain for DRX	$\varepsilon_{cr\_DRX}=p_{1}D_{0}^{p_{2}}Z^{p_{3}}$	(3)
DRX volume fraction	$X_{DRX}=1-\exp\left[-p_{7}{\left(\frac{\varepsilon -\varepsilon_{cr\_DRX}}{\varepsilon_{s}-\varepsilon_{cr\_DRX}}\right)}^{p_{8}}\right]$	(4)
Saturation strain	$\varepsilon_{s}=p_{4}D_{0}^{p_{5}}Z^{p_{6}}$	(5)
Grain size after DRX	$D_{DRX}=p_{9}Z^{-p_{10}}$	(6)
Grain growth	$D_{t+\Delta t}^{q}=D_{t}^{q}+K\;t\;\exp\left(-\frac{Q_{GROWTH}}{RT}\right)\;$	(7)

**Table 2:** Equations in the phase transformation models.

Parameter	Equation	
Incubation time of perlite for the remaining transformation	$\begin{array}{l} \tau_{P}=\frac{a_{9}D_{\gamma }^{a_{28}}}{{\left(\Delta T\right)}^{a_{11}}}\exp\left(\frac{a_{10}}{RT}\right)\\ \Delta T=A_{e1}-T \end{array}$	(9)
Incubation time of bainite for the remaining transformation	$\begin{array}{l} \tau_{b}=\frac{a_{17}D_{\gamma }^{a_{30}}}{{\left(\Delta T\right)}^{a_{19}}}\exp\left(\frac{a_{18}}{RT}\right)\\ \Delta T=B_{s}-T \end{array}$	(10)
Coefficients k for ferrite	$\begin{array}{l} k_{f}=\frac{a_{5}}{D_{\gamma }}\exp\left[-{\left(\frac{\left|\Delta T\right|}{a_{7}}\right)}^{a_{8}}\right]\\ \Delta T=T-T_{nose}\\ T_{nose}=A_{e3}-\frac{400}{D_{\gamma }}+a_{6} \end{array}$	(11)
Coefficients k for perlite	$\begin{array}{l} k_{p}=\frac{a_{15}}{D_{\gamma }^{a_{14}}}\exp\left[-{\left(\frac{\left|\Delta T\right|}{a_{13}}\right)}^{a_{8}}\right]\\ \Delta T=T-a_{12} \end{array}$	(12)
Coefficients k for bainite	$\begin{array}{l} k_{b}=\frac{a_{23}}{D_{\gamma }^{a_{29}}}\exp\left[-{\left(\frac{\left|\Delta T\right|}{a_{22}}\right)}^{a_{24}}\right]\\ \Delta T=T-a_{21} \end{array}$	(13)
Average carbon content in austenite	$c_{\gamma }=\frac{\left[c_{0}-\left[F_{f}+F_{b}\left(1-p\right)\right]c_{\alpha }\right]}{1-F_{f}-F_{b}\left(1-p\right)}$	(14)
Bainite start temperatures	$B_{s}=a_{20}$	(15)
Martensite start temperatures	$M_{s}=a_{25}-a_{26}c_{\gamma }$	(16)
Martensite volume fraction	$F_{m}=\left(1-F_{f}-F_{p}-F_{b}\right)\left\{1-\exp\left[-a_{27}\left(M_{s}-T\right)\right]\right\}$	(17)

**Table 3:** Chemical composition of the investigated steels, wt%.

Steel	C	Mn	Si	Cr	Ni	Cu	Mo
A	0.42	0.744	0.237	1.049	0.122	0.209	0.171
B	0.18	0.58	0.17	0.39	0.1	0.24	0.02
C	0.48	0.58	0.23	0.12	0.16	-	0.04

**Table 4:** Strains (*ε*) and temperatures (*T*, °C) in subsequent passes of physical simulation.

Part	Point	Pass 1		Pass 2		Pass 3		Pass 4	
		ε	*T*, °C	*ε*	*T*, °C	*ε*	*T*, °C	*ε*	*T*, °C
Adapter	P	0.48	1229	0.52	1235	0.1	1232	-	-
	Q	0.59	1228	0.89	1221	0.23	1211	-	-
Flange	P	0.4	1100	0.99	1103	0.07	1115	-	-
	Q	0.64	1099	0.87	1106	0.06	1103	-	-
Fork	P	0.11	1125	0.95	1120	0.39	1101	0.3	1093
	Q	0.12	1148	0.99	1147	1.69	1150	0.17	1120

**Table 5:** Coefficients in microstructure equations for the investigated steels.

Coefficient	Steel A	Steel B	Steel C
*n*	1.3025	1.4919	0.86
*a*_0_	2.814 × 10^−15^	9.9684 × 10^−13^	2.199 × 10^−16^
*a*_1_	−1.4016	−0.73206	−1.0784
*a*_2_	−0.1195	−0.15703	−0.42
*a*_3_	2.1793	3.9289	3.597
*Q_SRX_*	244080	92147	186,000
*b*_0_	0.6534	0.6143	28.713
*b*_1_	−0.2661	−0.1017	−0.3
*b*_2_	−0.06558	−0.013	−0.0796
*b*_3_	1.1471	1.1683	0.1746
*Q_DSRX_*	10,002	5008	6446
*p*_1_	0.61928 × 10^−3^	1.9337 × 10^−3^	0.60634 × 10^−4^
*p*_2_	0.2871	0.092	0.79995
*p*_3_	0.1906	0.1814	0.18753
*p*_4_	2.433 × 10^−3^	0.51143 × 10^−3^	0.5267 × 10^−5^
*p*_5_	0.1481	0.5252	1.476
*p*_6_	0.1951	0.1865	0.19823
*p*_7_	−1.4869	−1.159	−1.66863
*p*_8_	1.8849	1.5158	1.62773
*p*_9_	6822.71	3553	7676
*p*_10_	−0.1934	−0.1837	−0.21461
*Q_DEF_*	284,798	278,878	280,204
*q*	8.34	7	5
*K*	4.3322 × 10^30^	4 × 10^34^	2.837 × 10^16^
*Q_GROWTH_*	412,430	580,000	302,086

**Table 6:** Optimal coefficients in the phase transformation model for steel A.

*a*_4_	*a*_5_	*a*_6_	*a*_7_	*a*_8_	*a*_9_	*a*_10_	*a*_11_	*a*_12_
0.5417	0.74798	124.39	111.87	2.8367	6151.89	13.7849	1.118	666.76
*a*_13_	*a*_14_	*a*_15_	*a*_16_	*a*_17_	*a*_18_	*a*_19_	*a*_20_	*a*_21_
350	1.072	2.1496	0.05	7.185	76.43	3.4748	574.998	300
*a*_22_	*a*_23_	*a*_24_	*a*_25_	*a*_26_	*a*_27_	*a*_28_	*a*_29_	*a*_30_
236.55	10.2419	0.5	817.32	1299.8	0.011	0.0016	1.06559	1.2618

**Table 7:** Optimal coefficients in the phase transformation model for steel B.

*a*_4_	*a*_5_	*a*_6_	*a*_7_	*a*_8_	*a*_9_	*a*_10_	*a*_11_	*a*_12_
3.0	9.676	205.7	22.98	1.326	16.000	35.06	3.5	517
*a*_13_	*a*_14_	*a*_15_	*a*_16_	*a*_17_	*a*_18_	*a*_19_	*a*_20_	*a*_21_
119	0.364	8.4	0.291	0.0167	9.804	0.168	569.8	378.7
*a*_22_	*a*_23_	*a*_24_	*a*_25_	*a*_26_	*a*_27_	*a*_28_	*a*_29_	*a*_30_
61.66	10.86	0.5	362.1	0.047	0.011	0.516	0.636	0.927

**Table 8:** Optimal coefficients in the phase transformation model for steel C.

*a*_4_	*a*_5_	*a*_6_	*a*_7_	*a*_8_	*a*_9_	*a*_10_	*a*_11_	*a*_12_
1.33	5.896	185.7	75.85	2.713	16.000	12.54	3.5	594.9
*a*_13_	*a*_14_	*a*_15_	*a*_16_	*a*_17_	*a*_18_	*a*_19_	*a*_20_	*a*_21_
47.29	0.664	18.19	0.487	0.309	0.012	0.1	535.4	498.13
*a*_22_	*a*_23_	*a*_24_	*a*_25_	*a*_26_	*a*_27_	*a*_28_	*a*_29_	*a*_30_
15.63	13.2	3.7	656.7	696.4	0.011	1.3975	0.549	0.045
